# HLA-A, -B, -C, -DRB1 and -DQB1 allele and haplotype frequencies in Lebanese and their relatedness to neighboring and distant populations

**DOI:** 10.1186/s12864-022-08682-7

**Published:** 2022-06-20

**Authors:** Wassim Y. Almawi, Rita Nemr, Ramzi R. Finan, F. Lisa Saldhana, Abdelhafidh Hajjej

**Affiliations:** 1 Faculte’ Des Sciences de Tunis, Universite’ de Tunis El Manar, Manar II, 2092 Tunis, Tunisia; 2 School of Medicine, Lebanese American University, Beirut, Lebanon; 3 Faculty of Medicine, Universite’ St. Joseph, Beirut, Lebanon; 4 Department of Immunogenetics, National Blood Transfusion Center, Tunis, Tunisia

**Keywords:** Alleles, Genotypes, Haplotypes, Human Leukocyte Antigens, Lebanon

## Abstract

**Background:**

This study examined the origin of present-day Lebanese using high-resolution HLA class I and class II allele and haplotype distributions. The study subjects comprised 152 unrelated individuals, and their HLA class I and class II alleles and two-locus and five-locus haplotypes were compared with those of neighboring and distant communities using genetic distances, neighbor-joining dendrograms, correspondence, and haplotype analyses. HLA class I (*A*, *B*, *C*) and class II (*DRB1*, *DQB1*) were genotyped at a high-resolution level by PCR-SSP.

**Results:**

In total, 76 alleles across the five HLA loci were detected: A*03:01 (17.1%), A*24:02 (16.5%), B*35:01 (25.7%), C*04:01 (25.3%), and C*07:01 (20.7%) were the most frequent class I alleles, while DRB1*11:01 (34.2%) and DQB1*03:01 (43.8%) were the most frequent class II alleles. All pairs of HLA loci were in significant linkage disequilibrium. The most frequent two-locus haplotypes recorded were DRB1*11:01 ~ DQB1*03:01 (30.9%), B*35:01-C*04:01 (20.7%), B*35:01 ~ DRB1*11:01 (13.8%), and A*24:02 ~ B*35:01 (10.3%). Lebanese appear to be closely related to East Mediterranean communities such as Levantines (Palestinians, Syrians, and Jordanians), Turks, Macedonians, and Albanians. However, Lebanese appear to be distinct from North African, Iberian, and Sub-Saharan communities.

**Conclusions:**

Collectively, this indicates a limited genetic contribution of Arabic-speaking populations (from North Africa or the Arabian Peninsula) and Sub-Saharan communities to the present-day Lebanese gene pool. This confirms the notion that Lebanese population are of mixed East Mediterranean and Asian origin, with a marked European component.

**Supplementary Information:**

The online version contains supplementary material available at 10.1186/s12864-022-08682-7.

## Background

The Human Leukocyte Antigen (HLA) system is among the most polymorphic systems in mammals. As of January 23, 2019, 21,499 (15,586 class I and 5,913 class II) alleles of the HLA genes have been reported, of which the B locus with 5,881 alleles is the most polymorphic (http://hla.alleles.org). The HLA region lies on the short arm of chromosome 6 (6p21.3) and harbors in excess of 220 genes involved in diverse functions [1]. The presence of hundreds of genes within a 3.6-Mb distance leads to the bulk transmission of haplotypes due to linkage disequilibrium, defined as the nonrandom (preferential) association between alleles of close loci (http://hla.alleles.org). The HLA genes play a key role in the immune response [1] and the pathogenesis of mostly autoimmune diseases [2–4] and are very valuable tools in tracing the history of human migration due to the presence of linkage disequilibrium as well as allelic, genetic, and protein diversity [5, 6].

Lebanon is an East Mediterranean country and, with an area of 10,452 km^2^, is a small state in mainland Asia. The location of Lebanon at the crossroads of Asia, Europe, and Africa has contributed to its 5,000-year-old history and resulted in a distinct cultural identity marked by religious and ethnic diversity. Lebanon was home to the Phoenicians, who settled the country for almost 3,000 years but were then subject to a wave of invasion, starting with the Assyrians (the seventh century) invading Phoenicia, followed shortly by the Egyptians and, subsequently, Alexander the Great in the fourth century [7]. Following the division of the Roman Empire into the Western Empire and the Eastern Empire (Byzantium), Lebanon fell under Byzantine rule from 395 to 634 [7, 8]. Because of Arab conquest and the capture of Damascus in 635, Lebanon was ruled by the Umayyad (660–750), Abbasid (749–1258), and Fatimid (909–1171) dynasties and, later, by the Ottomans in 1516, who conquered most of present-day Middle East/North Africa until 1918 [7, 8]. The limited Crusades between 1096 and 1271 witnessed the introduction of European influence into Greater Syria (Lebanon, Syria, and Palestine) and the enforcement of Christianity in the mountain regions.

The population of Lebanon (est. 6,859,408) comprises descendants of diverse ethnicities who are either indigenous or have invaded and occupied Lebanon over the past six millennia. This linguistic, religious, and racial diversity is associated with significant admixture, making present-day Lebanon a mosaic of interrelated cultures. This paper investigates the HLA profile of the Lebanese population, which is compared to the profiles of neighboring and distant populations. It is the first to examine both class I and class II profiles and the first to identify common five-locus HLA haplotypes in the Lebanese population.

## Results

### HLA allele frequencies in the studied population

The distributions of the HLA class I (*HLA*-*A*, -*B*, and -*C*) and class II (*HLA-DRB1* and -*DQB1*) genotypes in all studied loci (Table 1) were within the Hardy–Weinberg Equilibrium (HWE) in the Lebanese participants. Table 1 shows the frequencies of the *HLA-A*, -*B*, -*C*, -*DRB1*, and -*DQB1* alleles detected in the studied population. In total, 76 HLA alleles were observed in the Lebanese. Among the *HLA-A* alleles, 17 were identified, of which A*03:01 (17.1%), A*24:02 (16.5%), and A*02:01 (14.5%) were the most frequent. Of the 25 *HLA-B* alleles identified, B*35:01 (25.7%) and B*18:01 (8.2%) were the most frequent. Fourteen *HLA-C* alleles were detected, the most frequent being C*04:01 (25.3%), C*07:01 (20.7%), and C*12:01 (14.5%). Thirteen *HLA-DRB1* alleles were also detected, of which DRB1*11:01 was the most frequent (34.2%), followed by DRB1*04:01 (12.8%) and DRB1*15:01 (11.5%). Lastly, DQB1*03:01 (43.8%) and DQB1*06:01 (16.5%) were the most common of the seven identified *DQB1* alleles. This was comparable to the distribution of Class I and Class II alleles in Europeans and Mediterranean populations.

**Table 1:** HLA-A, -B, -C -DRB1 and -DQB1 Allele Frequencies in Lebanese population (2n: 304)

HLA-A locus		HLA-B locus		HLA-C locus		HLA-DRB1 locus		HLA-DQB1 locus	
**Allele**	**Frequency**	**Allele**	**Frequency**	**Allele**	**Frequency**	**Allele**	**Frequency**	**Allele**	**Frequency**
03:01	0.171	35:01	0.257	04:01	0.253	11:01	0.342	03:01	0.438
24:02	0.164	18:01	0.082	07:01	0.207	04:01	0.128	06:01	0.164
02:01	0.145	08:01	0.059	12:01	0.145	15:01	0.115	05:01	0.158
01:01	0.128	44:02	0.056	06:02	0.099	03:01	0.082	02:01	0.151
11:01	0.086	14:02	0.049	03:02	0.059	13:01	0.082	03:02	0.049
32:01	0.063	49:01	0.049	15:02	0.056	07:01	0.072	03:03	0.020
23:01	0.046	52:01	0.049	08:01	0.039	01:01	0.049	04:01	0.020
30:01	0.046	07:02	0.043	16:04	0.033	14:01	0.049	**Total**	1.000
29:01	0.039	41:01	0.039	17:01	0.030	16:01	0.026	*P* (HWE)	0.154
33:01	0.039	51:02	0.036	12:02	0.026	10:01	0.023	χ^2^	2.04
68:01	0.023 0.0230	13:01	0.030	02:02	0.020	03:02	0.010		
69:01	0.016	50:01	0.030	05:01	0.020	08:01	0.010		
26:01	0.013	15:10	0.026	01:02	0.007	12:01	0.010		
31:01	0.007	38:01	0.026	14:02	0.007	**Total**	**1.000**		
66:02	0.007	40:20	0.026	**Total**	**1.000**	*P* (HWE)	0.332		
09:01	0.003	57:01	0.023	*P* (HWE)	0.555	χ^2^	0.94		
35:01	0.003	58:01	0.020	χ^2^	0.35				
**Total**	**1.000**	27:01	0.016						
*P* (HWE)	0.781	39:01	0.016						
χ^2^	0.08	45:01	0.016						
		53:01	0.016						
		55:01	0.013						
		73:01	0.013						
		37:01	0.004						
		42:01	0.004						
		**Total**	**1.000**						
		*P* (HWE)	0.849						
		χ^2^	0.04						

### Allelic comparison between the Lebanese and other populations

**Fig. 1:**
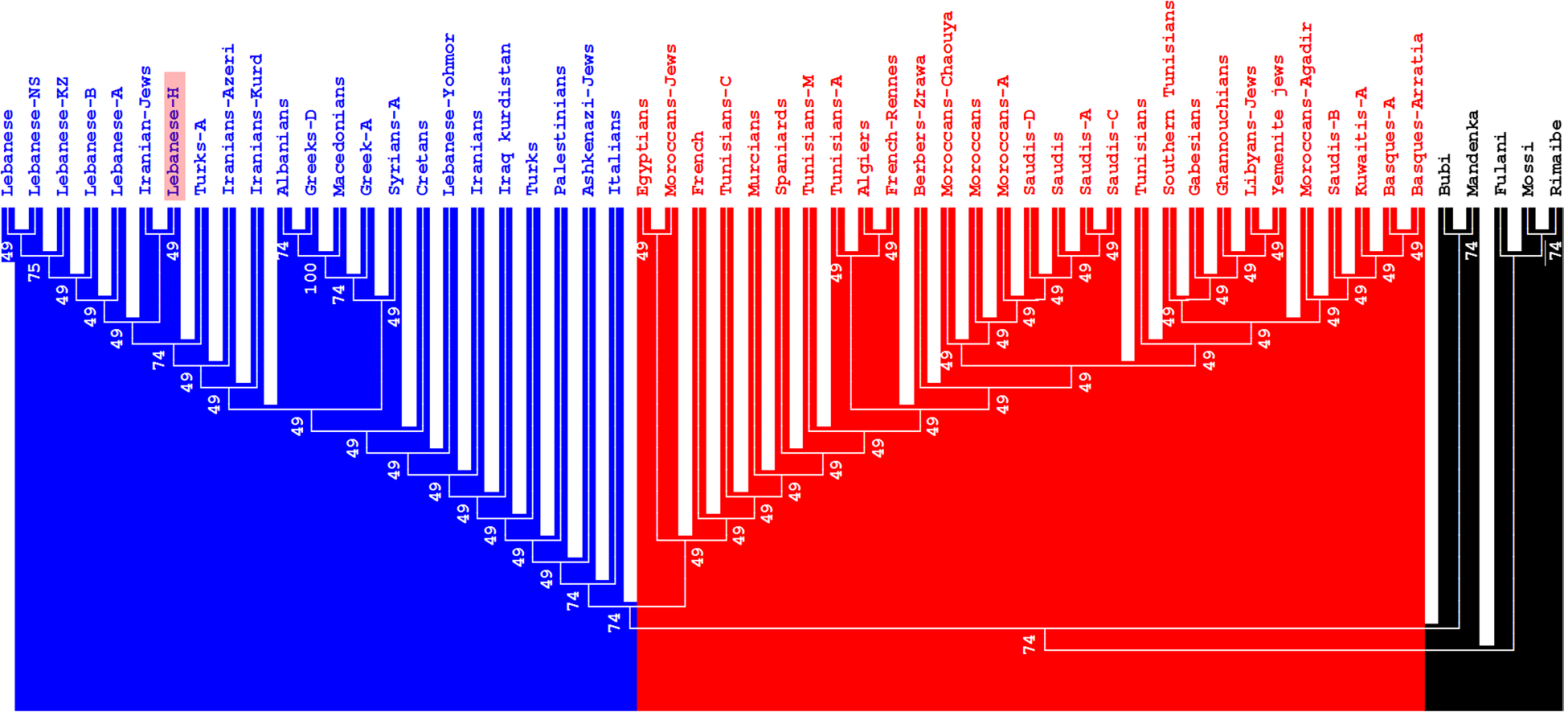
Neighbor-Joining dendrograms (UPGMA), based on Standard genetic distances (SGD), showing relatedness between Lebanese and other populations using HLA-DRB1 and -DQB1 allele frequencies data. Populations’ data were taken from references detailed in Supplementary Table 4. Bootstrap values from 1.000 replicates are shown

**Fig. 2:**
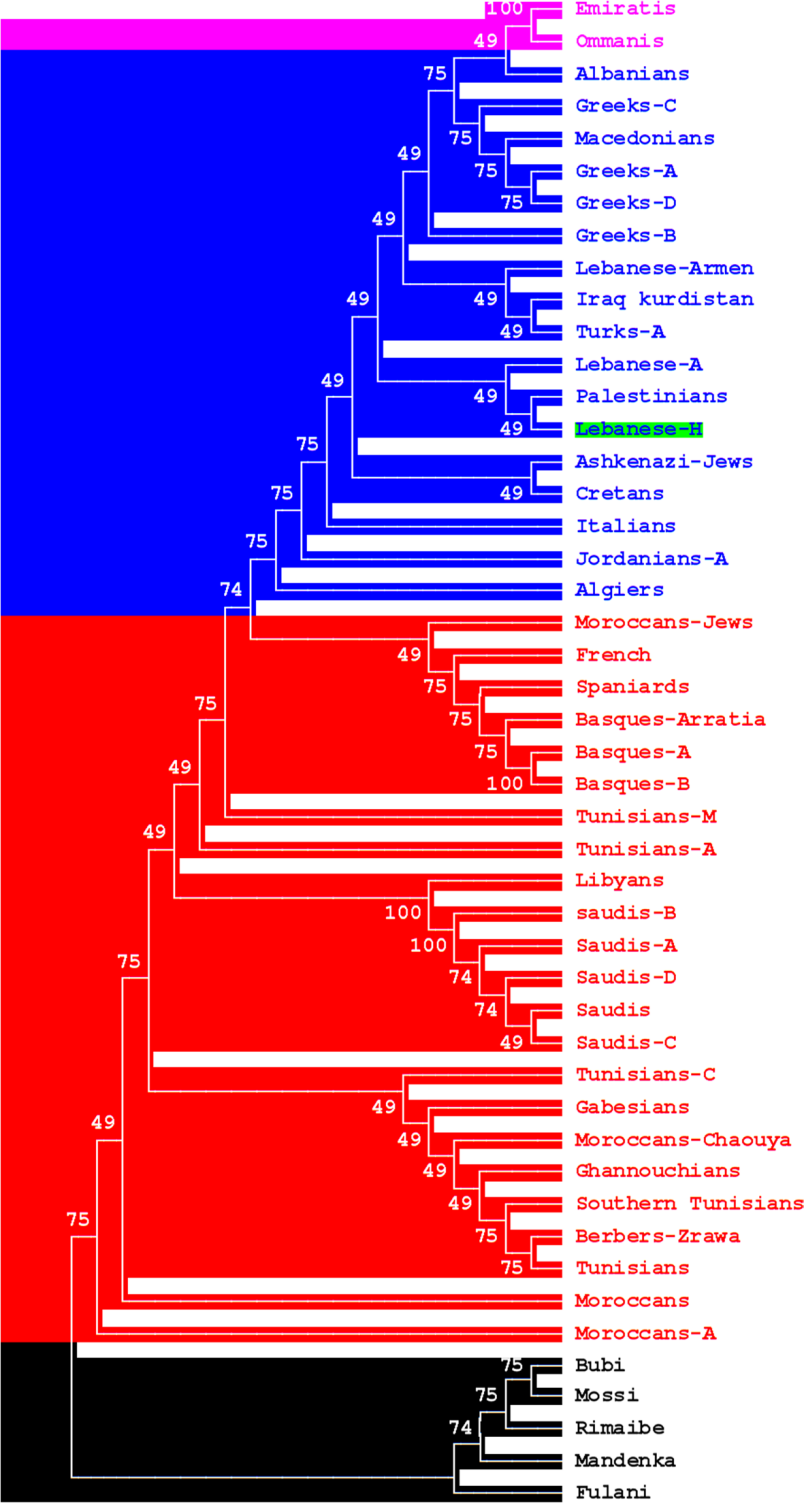
Neighbor-Joining dendrograms (UPGMA), based on Standard genetic distances (SGD), showing relatedness between Lebanese and other populations using generic HLA-A and -B allele frequencies data. Populations’ data were taken from references detailed in Supplementary Table 4. Bootstrap values from 1.000 replicates are shown

The differences in the typing methods between the study group and reference populations affected the data presentation, notably the calculation of the SGD and the comparison between the populations. The HLA profiles of the 152 Lebanese participants were compared to those of other Arabic-speaking, Mediterranean, and Sub-Saharan populations using high- and low-resolution HLA data; the latter were included because some reference populations lacked high-resolution data. Using *DRB1* and *DQB1* allele frequencies, standard genetic distance (SGD) analysis identified three clusters (Fig. 1). The first comprised East Mediterranean (pan-Lebanese, Palestinians, Greeks, Syrians, Cretans, Macedonians, Albanians, and Turks), Italians, Iranians, Iraqi Kurds, and Ashkenazi Jews. The second included Iberians, North Africans, Saudis, French, and Egyptians, while the third comprised Sub-Saharans (Bubi, Mandenka, Mossi, Fulani, and Rimaibe). NJ dendrograms identified three populations using SGD based on *HLA-A* and -*B* allele frequencies (Fig. 2). The first included East Mediterranean (pan-Lebanese, Palestinians, Cretans, Macedonians, Albanians, Greeks, and Turks), Iranians, Jordanians, Italians, Iraqi Kurds, and Ashkenazi Jews. The second contained Iberians, North Africans, Saudis, and French, while the third contained Sub-Saharans (Fig. 2).

**Fig. 3:**
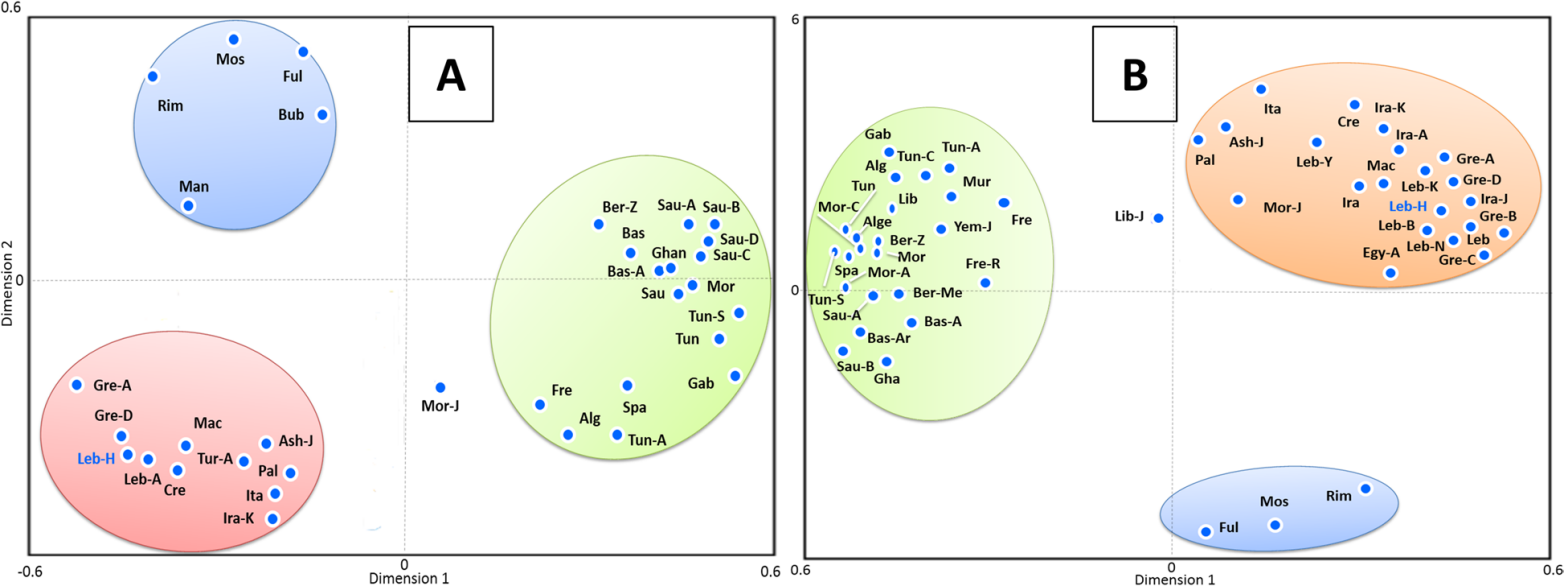
**A** Correspondence analysis (bi-dimensional representation), based on the standard genetic distances, showing the relationship between Lebanese and worldwide populations according to HLA-A, -B, DRB1, and DQB1 allele frequencies data. Populations data were taken from references detailed in Supplementary Table 5. **B** Correspondence analysis (bi-dimensional representation), based on the standard genetic distances, showing the relationship between Lebanese and worldwide populations according to High resolution (4-digits) HLA-DRB1 allele frequencies data. Populations data were taken from references detailed in Supplementary Table 4

Using *HLA-A*, -*B*, -*DRB1*, and -*DQB1* allele frequencies, SGD identified three clusters (Fig. 3A). The first comprised Iberians, French, North Africans, and Saudis. The second consisted of East Mediterranean (pan-Lebanese, Palestinians, Cretans, Macedonians, Greeks, and Turks), Italians, Iraqi Kurds, and Ashkenazi Jews, while the third contained Sub-Saharan populations (Bubi, Mandenka, Mossi, Fulani, and Rimaibe). Using high-resolution *DRB1* data, correspondence analysis depicted three clusters (Fig. 3B). The first grouped West Europeans (Iberians and French), North Africans, Saudis, and Yemenite Jews. The second combined East Mediterraneans (Palestinians, Cretans, Lebanese, Macedonians, and Greeks), Iranians, Italians, Iraqi Kurds, Egyptians, and Ashkenazi Jews, while the third comprised Sub-Saharan populations (Mossi, Fulani, and Rimaibe) (Fig. 3B).

SGDs between Lebanese and other populations showed an absence of clear discontinuities in terms of the genetic distances between the Lebanese-H and other populations (Supplementary Table 1). Based on the data of *A*, *B*, *DRB1*, and *DQB1* loci, SGDs confirmed that Lebanese-H are closer to East Mediterranean than West Mediterranean populations, but distant from Sub-Saharans. SGDs showed that Lebanese-A (8.2 × 10^–3^), Iraqi Kurds (2.8 × 10^–4^), Palestinians (4.1 × 10^–4^), Cretans (4.9 × 10^–4^), and Turks-A (5.8 × 10^–4^) have the closest genetic distances to the Lebanese-H. Collectively, this confirms the origin of present-day Lebanese compared to neighboring Mediterranean, Levantine, and European populations.

### *HLA-A*, -*B*, -*DRB1*, and -*DQB1* LD

The global linkage disequilibrium (LD) estimates of the associations between HLA loci are shown in Supplementary Table 2. All HLA loci pairs showed significant LD, with *C:B* (D’ = 0.82) and *DRB1*:*DQB1* (D’ = 0.80) having the strongest associations, and *C*:*DQB1* having the lowest value (D’ = 0.33). Intermediate LD estimates were seen in *A*:*B* (D’ = 0.59), *B*:*DRB1* (D’ = 0.56), *B*:*DQB1* (D’ = 0.52), and *A*:*C* (D’ = 0.50).

Two-locus HLA LD for the four pairs of loci with the highest LD values (*A* ~ *B*, *B* ~ *C*, *B* ~ *DRB1*, and *DRB1* ~ *DQB1*) was next determined (Table 2). The complete list of two-locus HLA haplotypes is found in Supplementary Table 3. Of the 15 *A* ~ *B* haplotypes with frequencies exceeding 1%, A*24:02 ~ B*35:01 (10.3%) was the most frequent, with the frequencies of the other *A* ~ *B* haplotypes varying between 1.1% and 2.8% (Table 2). Similarly, of the 12 most common *B* ~ *DRB1* haplotypes (frequencies > 1%), B*35:01 ~ DRB1*11:01 (13.8%) was the most frequent, while the frequencies of the other haplotypes did not exceed 4%. Furthermore, among the 17 common *B* ~ *C* haplotypes (frequencies > 1%), B*35:01 ~ C*04:01 (20.7%) was the most frequent, with the frequencies of the other haplotypes ranging from 1.3 to 5.6%. Lastly, the most frequent *DRB1* ~ *DQB1* haplotype detected was DRB1*11:01 ~ DQB1*03:01 (30.9%), followed by DRB1*15:01 ~ DQB1*06:01 (10.5%) and DRB1*03:01 ~ DQB1*02:01 (7.9%) (Table 2).

**Table 2:** Frequent HLA Class I and Class II two-Locus haplotypes in Lebanese Study Subjects

HLA loci	Haplotype	Freq	D'	χ^2^	*P*	HLA Loci	Haplotype	Freq	D'	χ^2^	*P*
***A* ~ *B***	A*24:02 ~ B*35:01	0.103	0.44	34.68	< 1.0 × 10^–6^	***B*** ~ ***DRB1***	B*49:01 ~ DRB1*04:01	0.020	0.31	9.95	2.0 × 10^–3^
	A*24:02 ~ B*18:01	0.028	0.22	6.69	0.010		B*07:02 ~ DRB1*15:01	0.016	0.30	9.68	2.0 × 10^–3^
	A*02:01 ~ B*41:01	0.020	0.47	14.35	1.5 × 10^–6^		B*13:01 ~ DRB1*07:01	0.016	0.52	32.25	< 1.0 × 10^–6^
	A*02:01 ~ B*40:20	0.020	0.71	23.67	1.0 × 10^–6^		B*14:02 ~ DRB1*04:01	0.016	0.26	6.53	0.010
	A*02:01 ~ B*08:01	0.020	0.27	6.93	8.0 × 10^–3^		B*15:10 ~ DRB1*15:01	0.016	0.58	20.96	5.0 × 10^–6^
	A*11:01 ~ B*52:01	0.020	0.26	12.20	5.0 × 10^–4^		B*41:01 ~ DRB1*03:01	0.016	0.36	18.51	1.7 × 10^–7^
	A*11:01 ~ B*14:02	0.018	0.17	5.61	0.018		B*51:02 ~ DRB1*13:01	0.016	0.40	20.96	5.0 × 10^–6^
	A*32:01 ~ B*44:02	0.018	0.33	28.99	< 1.0 × 10^–6^		B*38:01 ~ DRB1*04:01	0.013	0.42	9.76	1.8 × 10^–5^
	A*03:01 ~ B*38:01	0.016	0.55	12.12	5.0 × 10^–4^	***B* ~ *C***	B*35:01 ~ C*04:01	0.207	0.75	170.51	< 1.0 × 10^–6^
	A*01:01 ~ B*52:01	0.015	0.30	8.69	3.0 × 10^–3^		B*08:01 ~ C*07:01	0.056	0.93	63.29	< 1.0 × 10^–6^
	A*23:01 ~ B*50:01	0.014	0.41	30.08	< 1.0 × 10^–6^		B*18:01 ~ C*12:01	0.049	0.53	45.61	< 1.0 × 10^–6^
	A*29:01 ~ B*51:02	0.013	0.33	28.36	< 1.0 × 10^–6^		B*49:01 ~ C*07:01	0.046	0.91	50.63	< 1.0 × 10^–6^
	A*30:01 ~ B*13:01	0.013	0.42	32.86	< 1.0 × 10^–6^		B*52:01 ~ C*12:01	0.039	0.77	54.73	< 1.0 × 10^–6^
	A*68:01 ~ B*35:01	0.013	0.56	5.72	0.017		B*13:01 ~ C*06:02	0.030	1.00	84.70	< 1.0 × 10^–6^
	A*23:01 ~ B*49:01	0.012	0.27	18.80	1.5 × 10^–7^		B*38:01 ~ C*12:01	0.026	1.00	48.55	< 1.0 × 10^–6^
	A*33:01 ~ B*14:02	0.011	0.30	23.00	2.0 × 10^–6^		B*50:01 ~ C*06:02	0.026	0.88	65.11	< 1.0 × 10^–6^
***B*** ~ ***DRB1***	B*35:01 ~ DRB1*11:01	0.138	0.31	19.65	9.0 × 10^–6^		B*15:10 ~ C*07:01	0.023	0.82	22.30	2.0 × 10^–6^
	B*08:01 ~ DRB1*03:01	0.039	0.64	86.59	< 1.0 × 10^–6^		B*07:02 ~ C*15:02	0.020	0.43	42.32	< 1.0 × 10^–6^
	B*52:01 ~ DRB1*15:01	0.039	0.77	72.64	< 1.0 × 10^–6^		B*40:20 ~ C*03:02	0.020	0.73	70.38	< 1.0 × 10^–6^
	B*18:01 ~ DRB1*01:01	0.020	0.35	21.11	4.0 × 10^–6^		B*41:01 ~ C*17:01	0.016	0.54	65.15	< 1.0 × 10^–6^
***B* ~ *C***	B*51:02 ~ C*16:04	0.016	0.48	63.78	< 1.0 × 10^–6^	***DRB1*** ~ ***DQB1***	DRB1*13:01 ~ DQB1*06:01	0.049	0.52	37.60	< 1.0 × 10^–6^
	B*44:02 ~ C*05:01	0.013	0.79	53.31	< 1.0 × 10^–6^		DRB1*04:01 ~ DQB1*03:02	0.046	0.92	81.44	< 1.0 × 10^–6^
	B*44:02 ~ C*16:04	0.013	0.36	23.19	< 1.0 × 10^–6^		DRB1*07:01 ~ DQB1*02:01	0.046	0.57	43.45	< 1.0 × 10^–6^
	B*53:01 ~ C*04:01	0.013	0.73	8.03	4.0 × 10^–3^		DRB1*14:01 ~ DQB1*05:01	0.046	0.92	71.35	< 1.0 × 10^–6^
	B*57:01 ~ C*07:01	0.013	0.46	5.78	0.016		DRB1*01:01 ~ DQB1*05:01	0.043	0.84	59.61	< 1.0 × 10^–6^
***DRB1*** ~ ***DQB1***	DRB1*11:01 ~ DQB1*03:01	0.309	0.83	139.70	< 1.0 × 10^–6^		DRB1*16:01 ~ DQB1*05:01	0.026	1.00	43.82	< 1.0 × 10^–6^
	DRB1*15:01 ~ DQB1*06:01	0.105	0.90	161.82	< 1.0 × 10^–6^		DRB1*10:01 ~ DQB1*05:01	0.023	1.00	38.21	< 1.0 × 10^–6^
	DRB1*03:01 ~ DQB1*02:01	0.079	0.95	138.72	< 1.0 × 10^–6^						

### Class I and class II extended haplotype analysis

Extended class I and class II haplotype analysis using 304 chromosomes from the 152 subjects identified 198 five-locus haplotypes (Table 3), showing the most common extended haplotypes detected in the Lebanese. The most frequent (> 0.9%) five-locus *A* ~ *B* ~ *C* ~ *DRB1* ~ *DQB1* haplotype was A*24:02 ~ B*35:01 ~ C*04:01 ~ DRB1*11:01 ~ DQB1*03:01 (5.3%), followed by A*02:01 ~ B*52:01 ~ C*12:01 ~ DRB1*15:01 ~ DQB1*06:01 (2.3%) and A*24:02 ~ B*18:01 ~ C*12:01 ~ DRB1*11:01 ~ DQB1*03:01 (2.3%).

**Table 3:** Frequent five- locus HLA haplotypes in Lebanese population

HLA 5-Locus Haplotype	Frequency
A*24:02 ~ B*35:01 ~ C*04:01 ~ DRB1*11:01 ~ DQB1*03:01	5.26 × 10^–2^
A*02:01 ~ B*52:01 ~ C*12:01 ~ DRB1*15:01 ~ DQB1*06:01	2.30 × 10^–2^
A*24:02 ~ B*18:01 ~ C*12:01 ~ DRB1*11:01 ~ DQB1*03:01	2.30 × 10^–2^
A*01:01 ~ B*35:01 ~ C*04:01 ~ DRB1*11:01 ~ DQB1*05:01	1.65 × 10^–2^
A*02:01 ~ B*35:01 ~ C*04:01 ~ DRB1*11:01 ~ DQB1*03:01	1.65 × 10^–2^
A*03:01 ~ B*08:01 ~ C*07:01 ~ DRB1*03:01 ~ DQB1*02:01	1.65 × 10^–2^
A*24:02 ~ B*35:01 ~ C*12:01 ~ DRB1*04:01 ~ DQB1*03:02	1.65 × 10^–2^
A*02:01 ~ B*08:01 ~ C*07:01 ~ DRB1*03:01 ~ DQB1*02:01	1.32 × 10^–2^
A*32:01 ~ B*35:01 ~ C*04:01 ~ DRB1*07:01 ~ DQB1*03:01	1.32 × 10^–2^
A*01:01 ~ B*52:01 ~ C*12:01 ~ DRB1*15:01 ~ DQB1*06:01	9.87 × 10^–3^
A*01:01 ~ B*57:01 ~ C*07:01DRB1*11:01 ~ DQB1*03:01	9.87 × 10^–3^
A*02:01 ~ B*38:01 ~ C*03:02 ~ DRB1*04:01 ~ DQB1*06:01	9.87 × 10^–3^
A*03:01 ~ B*35:01 ~ C*04:01 ~ DRB1*11:01 ~ DQB1*02:01	9.87 × 10^–3^
A*11:01 ~ B*35:01 ~ C*04:01 ~ DRB1*14:01 ~ DQB1*05:01	9.87 × 10^–3^
A*11:01 ~ B*44:02 ~ C*04:01 ~ DRB1*15:01 ~ DQB1*06:01	9.87 × 10^–3^
A*11:01 ~ B*55:01 ~ C*03:02 ~ DRB1*16:01 ~ DQB1*03:01	9.87 × 10^–3^
A*24:02 ~ B*35:01 ~ C*04:01 ~ DRB1*04:01 ~ DQB1*03:01	9.87 × 10^–3^
A*32:01 ~ B*44:02 ~ C*05:01 ~ DRB1*11:01 ~ DQB1*03:01	9.87 × 10^–3^
A*03:01 ~ B*50:01 ~ C*06:02 ~ DRB1*11:01 ~ DQB1*03:01	9.42 × 10^–3^
A*23:01 ~ B*49:01 ~ C*07:01 ~ DRB1*11:01 ~ DQB1*03:01	9.42 × 10^–3^

### EWH test of neutrality

The results of the EWH test for the five HLA loci in the Lebanese are shown in Supplementary Table 4. No significant deviation was found for *B* (*P* = 0.203), *C* (*P* = 0.073), *DRB1* (*P* = 0.180), or *DQB1* (*P* = 0.102) loci; significant deviation was observed only for the *A* locus (*P* = 0.025). Negative *F*nd values were recorded for the analyzed loci, and the homozygosity was lower than expected under selective neutrality. Significant differences were noted between the observed and expected homozygotes for the *DRB1* (*P* = 0.033) and *DQB1* (*P* = 0.019) loci, indicating an overall trend away from the null hypothesis of neutral evolution, suggesting that the allele frequency distributions at all loci were shaped by balancing selection.

### Genetic admixture in Lebanese

The estimation of the genetic contribution rates to the Lebanese was performed using A, B, DRB1, and DQB1 loci from parental populations from Italy (Europe), Pakistan (Asia), Morocco (North Africa), and Mossi (Sub-Saharan Africa) (Table 4). The most notable contribution was seen from Europeans (0.8434 – 1.0742), followed by Asians (0.1566 – 0.2070). The North African and Sub-Saharan contributions to the Lebanese genetic pool were low, as indicated by the negative value of the admixture coefficient established for Mossi (–0.1117 – -0.0273) and Moroccans (-0.2539). Similar results were found regardless of the selected population (Sub-Saharan or North African).

**Table 4:** Genetic admixture in the Lebanese population

Parental populations	Admixture coefficient
Europeans, Asians	0.8434, 0.1566
Europeans, Asians, Sub-Saharans	0.9167, 0.1951, -0.1117
Europeans, Asians, Sub-Saharans, North Africans	1.0742, 0.2070, -0.0273, -0.2539

The populations used to calculate the genetic contribution from North Africa, Asia, Sub-Saharan Africa and Europe are, respectively, Moroccans, Pakistanis, Mossi, and Italians

## Discussion

Previous reports on the HLA profile of Lebanese focused on class II (DRB1 and DQB1) alleles and haplotype analyses, wherein statistical and anthropological analyses were virtually absent [5, 9–11]. This present work used the molecular data of both class I (*A*, *B*, *C*) and class II (*DRB1*, *DQB1*) loci in examining the possible origin of present-day Lebanese by analyzing the obtained results from a historical context. Using high-resolution molecular typing, 76 alleles were detected. However, allelic comparison of Lebanese to neighboring and distant populations was not always useful in view of the scarcity or absence of high-resolution data (six digits), mostly in neighboring populations. This limited the comparison to lower-resolution (four digits) levels.

The most common alleles among Lebanese are typically Mediterranean. For example, A*03:01 in the Lebanese participants (17.1%) was found at comparable frequencies in Czechs (18.9%), Croatians (11.8%), Belgians (17.1%), Germans (15.9%), and Georgians (13.8%). Higher frequencies of the A*03:01 allele were reported in Scandinavians such as Swedes (31.3%) and Finns (25.0%) (http://www.allelefrequencies.net). Moreover, A*24:02 (16.5% in Lebanese) was also found at comparable frequencies in Croatians (16.0%), Greek (11.8%), Iranian Kurds (17.6%), Italians (12.2%), and Romanians (12.7%) (http://www.allelefrequencies.net). It should be noted that A*24:02 is very frequent in China, Malaysia, Taiwan, and Japan. A*02:01, another common allele among the Lebanese (14.47%), is also frequent in Moroccans from Metalsa (17.8%), Bulgarians (30.0%), Saudis (13.6%) [Excoffier and Slatkin, 1995], Libyans (15.7%) [12], and Iranians (20.2%) [13].

Furthermore, B*35:01 was the most frequent *HLA-B* allele in the Lebanese (25.7%), and among the highest in the Mediterranean region. It has been reported to also be high in Iranian Kurds (22.0%), Italians (13.3%), and Romanians (10.1%) (http://www.allelefrequencies.net). Among the *HLA-C* loci, C*04:01 (25.3%) was the most common allele and is frequent in Iranian Balochs (28.6%) [13], Greeks (19.3%), and Italians (18.8%) (http://www.allelefrequencies.net). Moreover, C*07:01 (20.7%) was the second most common allele in the Lebanese participants and has also been reported for South Italian (20.6%), Greek (18.1%), Tunisian (12.6%), and Turkish (12.3%) populations (http://www.allelefrequencies.net). Furthermore, of the *HLA-DRB* alleles identified, DRB1*11:01 (34.2%) was frequent in the Lebanese and is also found at high frequencies in Iranians (21.9%), North Italians (20.5%), and Iranian Kurds (19.1%) (http://www.allelefrequencies.net). In addition, DQB1*03:01 (43.8%) was the most frequent *HLA*-*DQB1* allele in the Lebanese and has also been observed at high frequencies in Lebanese from Niha el Shouff (45.1%), Macedonians (35.0%), and Italians (34.9%) (http://www.allelefrequencies.net).

As their genes are separated by a reduced physical distance (PD) of 0.1 Mb, the *C*:*B* (D’ = 0.8179) and *DRB1*:*DQB1* (D’ = 0.7971) loci pairs had the highest LD values as compared to the *C*:*DQB1* pair, which had the weakest association (D’ = 0.3343), resulting from the larger PD separating the *C* and *DQB1* genes, which promotes an increased recombination rate. This was reminiscent of earlier studies, which documented that the D’ values are inversely proportional to the PD separating the two loci, as the recombination rate increases with the PD [14, 15]. The higher LD value obtained for *A*:*B* (D’ = 0.6031; PD = 1.4 Mb) compared to *B*:*DQB1* (D’ = 0.5234; PD = 1.24 Mb) and *A*:*C* (D’ = 0.4974; PD = 1.3) was attributed to the existence of recombination hot spots between specific HLA genes and/or the low levels of polymorphism seen at *C* and *DQB1* loci relative to *A* and *B* loci. Furthermore, negative *F*nd values were seen for all loci, indicating an overall direction toward balancing selection. This was in agreement with an earlier study documenting balancing selection in *A*, *C*, *B*, *DRB1*, *DQA1*, and *DQB1* HLA loci, with *DQA1* displaying the strongest [16].

Here, A*24:02 ~ B*35:01, B*35:01 ~ DRB1*11:01, B*35:01 ~ C*04:01, and DRB1*11:01 ~ DQB1*03:01 were frequent two-locus haplotypes in the Lebanese participants. While A*24:02 ~ B*35:01 has been reported for Romanians (1.5%) and Taiwanese (1.8%), B*35:01 ~ C*04:01 is frequent in Irish (5.2%), Italian (4.9%), Tunisian (4.0%), and Malian (7.7%) populations (http://www.allelefrequencies.net). Furthermore, B*35:01 ~ DRB1*11:01 is also seen in Italians (1.02%), while DRB1*11:01 ~ DQB1*03:01 is a frequent two-locus haplotype in Iranians (18.5%), Germans (14.4%), Italians (5.4%), and Tunisians (7.0%) (http://www.allelefrequencies.net). The high D' values and *DRB1* ~ *DQB1* and *B* ~ *C* haplotype frequencies (compared to the *B* ~ *DRB1* and *A* ~ *B* haplotypes) in the Lebanese were attributed to the reduced PDs between the *B* ~ *C* and *DQB1* ~ *DRB1* loci, resulting in decreased recombination between these genes. Furthermore, the most frequent extended haplotype (A*24:02 ~ B*35:01 ~ C*04:01 ~ DRB1*11:01 ~ DQB1*03:01; 5.3%) has been reported in its two-field form (A*24:02 ~ B*35:01 ~ C*04:01 ~ DRB1*11:01 ~ DQB1*03:01) in Turkish (0.2%) and Italian German minorities (0.04%) and Indian (0.5%) populations (http://www.allelefrequencies.net). In addition, A*02:01 ~ B*52:01 ~ C*12:01:01 ~ DRB1*15:01 ~ DQB1*06:01 and A*24:02 ~ B*18:01 ~ C*12:01 ~ DRB1*11:01 ~ DQB1*03:01 frequencies in Lebanese are the highest reported for any population.

Our analysis showed that the Lebanese participants were closely related to East Mediterranean (Turks, Albanians, Macedonians, Greeks, and Cretans), Levantine Arab (Syrians, Jordanians, and Palestinians), and Mesopotamian (Iraqis) populations. This can be explained by the fact that East Mediterranean countries share, with slight differences, a similar history and the same territory [17]. The Eastern Mediterranean Basin was historically characterized by high migratory flow between its sub-regions in all directions and in different periods (Greeks, Romans, and Ottomans). This favored admixture, reduced distances, and homogenized the Great Levant populations. The relatedness between the Levantine Arab populations is attributed to their close geographical proximity, which constituted one territory before the nineteenth-century British and French colonization. It is also attributed to their common ancient Canaanite ancestry, originating from East Africa or the Arabian Peninsula via Egypt in 3300 BC [18] and settling in the Levantine lowlands following the collapse of the Ghassulian civilization in 3800–3350 BC [19].

Based on data from *A*, *B*, *DRB1*, and *DQB1* loci, admixture analysis showed that most (up to 84%) of the genetic contribution to the Lebanese gene pool is derived from Europeans, with low genetic contributions from other regions, including the Arabian Peninsula, suggesting a low contribution of Arabs and Sub-Saharans to the Lebanese gene pool. This is in accord with the other analyses carried out in this work. Using high-resolution data, the analysis of the five HLA loci confirmed that the Lebanese are distant from North African (Tunisians, Moroccans, and Algerians), Iberian (Basques, Murcians, and Spaniards), and Arabian Peninsula (Saudis, Kuwaitis, and Emiratis) populations. This suggests a lack of contribution of North African and Arabian Peninsula populations to the gene pool of the Lebanese despite the Phoenicians’ invasion and long colonization of North Africa and the Arab conquest of Lebanon from as early as the seventh century, prompting speculation of "elite colonization" [20].

## Conclusions

In conclusion, our study based on NJ dendrograms, genetic distances, LD, admixture, and correspondence analyses showed that the Lebanese are related to Levantines, Eastern Mediterraneans, and Mesopotamians but are distinct from North African, Iberian, Saudi, and Sub-Saharan communities. Our study has shortcomings, namely the relatively low sample size (152 subjects), and lack of genotyping for HLA-DP locus due to purely financial reasons as the typing kit used (SSP2L) handles DRB1 and DQB1 only. Future studies aimed at typing larger number of subjects and additional HLA loci (DPB1, DQA1) are planned. The contribution of Arab Muslims and Sub-Saharans to the Lebanese gene pool seems weak. The results of this work are consistent with those found in our previous studies [5, 14, 21–23].

## Methods

### Study subjects

The study subjects comprised 152 unrelated healthy Lebanese individuals of both sexes (90 males and 62 females), who were randomly collected from the five provinces and the six major religious groups of Lebanon. These comprised hospital and university staff, blood donors, and volunteers from the community. None of the study particiants suffered from any acute or chronic disease, including neurologic, cardiac, or metabolic diseases, and were not on any medication at the time of specimen collection. The individuals were subjected to HLA class I and class II high-resolution genotyping and phylogenetic calculations. The origins of the other populations included for comparative purposes are detailed in Supplementary Table 5. Written informed consent to participate in the study was obtained from all participants; the Research & Ethics committees of St. Marc Medical Center and St. Georges University Hospital approved the study protocol in accordance with the Declaration of Helsinki.

### HLA genotyping

The Qiagen mini-spin column extraction kit was used to extract genomic DNA from EDTA-anticoagulated venous blood according to the manufacturer's instructions (Qiagen, Hilden, Germany). Low-resolution HLA-A, HLA-B, HLA-C, HLA-DRB1, and DQB1 typing was performed using generic polymerase chain reaction with sequence-specific primers (PCR-SSP) kits (One Lambda, Thousand Oaks, CA), while high-resolution typing was performed by PCR-SSP using SSP1L (class I) and SSP2L (class II) HLA genotyping kits according to the manufacturer’s specifications (Luminex–One Lambda, Canoga Park, CA).

### Statistical analysis

Python for Population Genomics (version 0.7.0, http://www.pypop.org) was used to perform Hardy–Weinberg tests, HLA allele frequency gene counts, pairwise linkage disequilibrium (LD) estimates [24, 25], and Ewens–Watterson homozygosity (EWH) tests. A test of homozygosity was applied to each locus using Slatkin’s Monte Carlo implementation of the exact test [26, 27]. The LD between alleles, haplotype frequencies [28], level of significance (P), chi-squared test, and relative LD (D′) were determined by the Arlequin software, version 2.0.1 [29, 30]. The admixture proportions were estimated by the ADMIX95 program (www.genetica.fmed.edu.uy/software.htm) [31]. The three-dimensional correspondence analysis and bi-dimensional representation were carried out using the VISTA V7.2.8 software [32]. Correspondence analysis and neighbor-joining (NJ) trees were constructed [33] with standard genetic distances (SGDs) [34] using the DISPAN software [35].

## Supplementary Information

**Additional file 1: Supplementary Table 1** Standard genetic distances (SGD) between Lebanese and other populations.

**Additional file 2: Supplementary Table 2** Pairwise global linkage disequilibrium (LD) estimates.

**Additional file 3: Supplementary Table 3** Complete list of HLA two-Locus haplotypes in Lebanese.

**Additional file 4: Supplementary Table 4** Ewens-Watterson homozygosity test of neutrality.

**Additional file 5: Supplementary Table 5** Populations used in the present work.

## Data Availability

The data contained in this study are available at the Dryad Data Repository, and can be accessed at: Almawi, Wassim (2022), HLA class I and class II allelic profile of healthy Lebanese population, Dryad, Dataset, https://doi.org/10.5061/dryad.1vhhmgqw2.

## Abbreviations

EWH: Ewens–Watterson homozygosity
HLA: Human Leukocyte Antigen
HWE: Hardy–Weinberg Equilibrium
LD: Linkage disequilibrium
NJ: Neighbor-joining
PCR-SSP: Polymerase chain reaction with sequence-specific primers
SGD: Standard genetic distance
