# Supplementary material for: HLA-A, -B, -C, -DRB1 and -DQB1 allele and haplotype frequencies in Lebanese and their relatedness to neighboring and distant populations
Source: BMC Genomics. 2022 Jun 20;23:456. doi: 10.1186/s12864-022-08682-7 (PMC9208108; doi:10.1186/s12864-022-08682-7)
Supplement: Supplementary file 1 — Additional file 1: Supplementary Table 1 Standard genetic distances (SGD) between Lebanese and other populations. [file 12864_2022_8682_MOESM1_ESM.docx]

**Supplementary Table 1**

**Standard genetic distances (SGD) between Lebanese and other populations**

| **Population** | **SGD*** | **Population** | **SGD*** |
| --- | --- | --- | --- |
| Lebanese-A | 0.008 | Mandenka | 0.208 |
| Iraq kurdistan | 0.028 | Southern Tunisians | 0.228 |
| Palestinians | 0.041 | Moroccans | 0.230 |
| Cretans | 0.049 | Saudis | 0.261 |
| Turks-A | 0.058 | Ghannouchians | 0.291 |
| Italians | 0.061 | Saudis-C | 0.298 |
| Greeks-A | 0.063 | Saudis-A | 0.305 |
| Greeks-D | 0.066 | Saudis-D | 0.307 |
| Ashkenazi-jews | 0.087 | Saudis-B | 0.331 |
| Macedonians | 0.088 | Basques-Arratia | 0.361 |
| Tunisians-A | 0.122 | Basques | 0.382 |
| French | 0.133 | Berbers | 0.393 |
| Algiers | 0.142 | Bubi | 0.424 |
| Moroc-Jews | 0.149 | Rimaibe | 0.562 |
| Gabesians | 0.169 | Fulani | 0.580 |
| Tunisians | 0.205 | Mossi | 0.608 |
| Spanish | 0.208 |  |  |

* SGD based on HLA-A, B, DRB1, and DQB1 data.
