# Supplementary material for: HLA-A, -B, -C, -DRB1 and -DQB1 allele and haplotype frequencies in Lebanese and their relatedness to neighboring and distant populations
Source: BMC Genomics. 2022 Jun 20;23:456. doi: 10.1186/s12864-022-08682-7 (PMC9208108; doi:10.1186/s12864-022-08682-7)
Supplement: Supplementary file 2 — Additional file 2: Supplementary Table 2 Pairwise global linkage disequilibrium (LD) estimates. [file 12864_2022_8682_MOESM2_ESM.docx]

**Supplementary Table 2**

Pairwise global linkage disequilibrium (LD) estimates

| **Locus pair** | **D'** | ***P*** |
| --- | --- | --- |
| *C:B* | 0.818 | <0.0001 |
| *DRB1:DQB1* | 0.797 | <0.0001 |
| *A:B* | 0.603 | <0.0001 |
| *B:DRB1* | 0.559 | <0.0001 |
| *B:DQB1* | 0.523 | <0.0001 |
| *A:C* | 0.497 | <0.0001 |
| *A:DRB1* | 0.470 | <0.0001 |
| *C:DRB1* | 0.399 | <0.0001 |
| *A:DQB1* | 0.393 | <0.0001 |
| *C:DQB1* | 0.334 | <0.0001 |

For D’, values above 0 show a positive association between loci; values above 0.5 indicate a very strong association.
