# Supplementary material for: HLA-A, -B, -C, -DRB1 and -DQB1 allele and haplotype frequencies in Lebanese and their relatedness to neighboring and distant populations
Source: BMC Genomics. 2022 Jun 20;23:456. doi: 10.1186/s12864-022-08682-7 (PMC9208108; doi:10.1186/s12864-022-08682-7)
Supplement: Supplementary file 3 — Additional file 3: Supplementary Table 3 Complete list of HLA two-Locus haplotypes in Lebanese. [file 12864_2022_8682_MOESM3_ESM.docx]

**Supplementary Table 3**

Complete list of HLA two-Locus haplotypes in Lebanese

| **Haplotype** | **Frequency** | **D’** | **χ^2^** | ***P*** | **Haplotype** | **Frequency** | **D’** | **χ^2^** | ***P*** |
| --- | --- | --- | --- | --- | --- | --- | --- | --- | --- |
| ***A~B*** |  |  |  |  | ***A~B*** |  |  |  |  |
| *01:01:01~52:01:01* | 0.0149 | 0.30 | 8.69 | 3.0 × 10^-3^ | *31:01:01~50:01:01* | 0.0033 | 0.48 | 14.01 | 1.8 × 10^-5^ |
| *01:01:01~73:01* | 0.0066 | 0.43 | 5.36 | 0.02 | *32:01~44:02:03* | 0.0178 | 0.33 | 28.99 | <1.0 ×10^-6^ |
| *02:01:01~40:20* | 0.0197 | 0.71 | 23.67 | <1.0 ×10^-6^ | *33:01~14:02* | 0.0115 | 0.30 | 23.00 | 2.0 × 10^-6^ |
| *02:01:01~41:01* | 0.0199 | 0.47 | 14.35 | 1.5 × 10^-6^ | *35:01~18:01:01* | 0.0033 | 1.00 | 10.12 | 1.5 × 10^-4^ |
| *02:01:01~08:01:01* | 0.0197 | 0.27 | 6.93 | 8.0 × 10^-3^ | *66:02~35:01:02* | 0.0066 | 1.00 | 6.07 | 0.01 |
| *03:01:01~38:01:01* | 0.0165 | 0.55 | 12.12 | 5.0 × 10^-4^ | *68:01:01~35:01:02* | 0.0132 | 0.56 | 5.72 | 0.02 |
| *03:01:01~27:01* | 0.0099 | 0.52 | 6.69 | 0.01 | *69:01:01~15:10* | 0.0033 | 0.23 | 8.32 | 4.0 × 10^-3^ |
| *11:01:01~14:02* | 0.0182 | 0.17 | 5.61 | 0.02 | *69:01:01~27:01* | 0.0033 | 0.24 | 12.32 | 4.0 × 10^-4^ |
| *11:01:01~15:10* | 0.0066 | 0.23 | 5.30 | 0.02 | ***B~DRB1*** |  |  |  |  |
| *11:01:01~52:01:01* | 0.0193 | 0.26 | 12.20 | 5.0 × 10^-4^ | *07:02:01~10:01:01* | 0.0099 | 0.40 | 26.05 | <1.0 ×10^-6^ |
| *11:01:01~55:01:01* | 0.0099 | 0.64 | 16.98 | 3.8 × 10^-6^ | *07:02:01~15:01:01* | 0.0164 | 0.30 | 9.68 | 2.0 × 10^-3^ |
| *23:01~49:01* | 0.0121 | 0.27 | 18.80 | 1.5 × 10^-6^ | *08:01:01~03:01:01* | 0.0395 | 0.64 | 86.59 | <1.0 ×10^-6^ |
| *23:01~42:01:01* | 0.0033 | 1.00 | 18.85 | 1.4 × 10^-6^ | *13:01:01~07:01:01* | 0.0164 | 0.52 | 32.25 | <1.0 ×10^-6^ |
| *23:01~50:01:01* | 0.0138 | 0.41 | 30.08 | <1.0 ×10^-6^ | *13:01:01~14:01:01* | 0.0066 | 0.18 | 5.91 | 0.02 |
| *24:02:01~18:01:01* | 0.0278 | 0.22 | 6.69 | 0.01 | *14:02~04:01:01* | 0.0164 | 0.26 | 6.53 | 0.01 |
| *24:02:01~35:01:02* | 0.1034 | 0.44 | 34.68 | <1.0 ×10^-6^ | *15:10~15:01:01* | 0.0164 | 0.58 | 20.96 | 5.0 × 10^-6^ |
| *26:01:01~18:01:01* | 0.0066 | 0.45 | 8.30 | 4.0 × 10^-3^ | *15:10~08:01:01* | 0.0033 | 0.31 | 11.15 | 8.0 × 10^-4^ |
| *26:01:01~58:01:01* | 0.0033 | 0.23 | 9.98 | 1.6 × 10^-4^ | *18:01:01~01:01:01* | 0.0197 | 0.35 | 21.11 | 4.0 × 10^-6^ |
| *29:01:01~07:02:01* | 0.0099 | 0.27 | 16.49 | 4.9 × 10^-6^ | *27:01~16:01:01* | 0.0066 | 0.38 | 27.70 | <1.0 ×10^-6^ |
| *29:01:01~51:02:01* | 0.0132 | 0.33 | 28.36 | <1.0 ×10^-6^ | *35:01:02~11:01:01* | 0.1382 | 0.31 | 19.65 | 9.0 × 10^-6^ |
| *30:01~45:01:01* | 0.0066 | 0.37 | 14.19 | 1.6 × 10^-5^ | *38:01:01~04:01:01* | 0.0132 | 0.42 | 9.76 | 1.8 × 10^-4^ |
| *30:01~13:01:01* | 0.0132 | 0.42 | 32.86 | <1.0 ×10^-6^ | *39:01:01~13:01:01* | 0.0099 | 0.56 | 18.06 | 2.1 × 10^-6^ |
| *31:01:01~51:02:01* | 0.0033 | 0.48 | 11.19 | 8.0 × 10^-4^ | *40:20~12:01:01* | 0.0033 | 0.49 | 17.63 | 2.7 × 10^-6^ |
| **Haplotype** | **Frequency** | **D’** | **χ^2^** | ***P*** | **Haplotype** | **Frequency** | **D’** | **χ^2^** | ***P*** |
| ***B~DRB1*** |  |  |  |  | ***B~C*** |  |  |  |  |
| *40:20~13:01:01* | 0.0099 | 0.32 | 9.33 | 2.0 × 10^-3^ | *38:01:01~12:01:01* | 0.0263 | 1.00 | 48.55 | <1.0 ×10^-6^ |
| *40:20~14:01:01* | 0.0066 | 0.21 | 7.05 | 8.0 × 10^-3^ | *39:01:01~08:01:01* | 0.0065 | 0.37 | 17.43 | 3.0 × 10^-5^ |
| *41:01~03:02:01* | 0.0033 | 0.30 | 6.90 | 9.0 × 10^-3^ | *40:20~03:02:01* | 0.0197 | 0.73 | 70.38 | <1.0 ×10^-6^ |
| *41:01~03:01:01* | 0.0164 | 0.36 | 18.51 | 1.7 × 10^-6^ | *40:20~15:02:01* | 0.0066 | 0.20 | 5.86 | 0.02 |
| *41:01~08:01:01* | 0.0033 | 0.30 | 6.90 | 9.0 × 10^-3^ | *41:01~02:02:01* | 0.0099 | 0.50 | 34.24 | <1.0 ×10^-6^ |
| *42:01:01~14:01:01* | 0.0033 | 1.00 | 19.33 | 1.1 × 10^-6^ | *41:01~17:01:01* | 0.0164 | 0.54 | 65.15 | <1.0 ×10^-6^ |
| *45:01:01~07:01:01* | 0.0099 | 0.57 | 21.08 | 4.0 × 10^-6^ | *42:01:01~17:01:01* | 0.0033 | 1.00 | 32.88 | <1.0 ×10^-6^ |
| *49:01~04:01:01* | 0.0197 | 0.31 | 9.95 | 2.0 × 10^-3^ | *44:02:03~05:01:01* | 0.0132 | 0.79 | 53.31 | <1.0 ×10^-6^ |
| *50:01:01~10:01:01* | 0.0066 | 0.26 | 14.39 | 1.5 × 10^-5^ | *44:02:03~16:04:01* | 0.0132 | 0.36 | 23.19 | <1.0 ×10^-6^ |
| *51:02:01~13:01:01* | 0.0164 | 0.40 | 20.96 | 5.0 × 10^-6^ | *49:01~07:01:01* | 0.0461 | 0.91 | 50.63 | <1.0 ×10^-6^ |
| *51:02:01~07:01:01* | 0.0099 | 0.21 | 6.82 | 9.0 × 10^-3^ | *50:01:01~06:02:01* | 0.0263 | 0.88 | 65.11 | <1.0 ×10^-6^ |
| *52:01:01~15:01:01* | 0.0395 | 0.77 | 72.64 | <1.0 ×10^-6^ | *51:02:01~14:02* | 0.0066 | 0.65 | 34.53 | <1.0 ×10^-6^ |
| *55:01:01~16:01:01* | 0.0099 | 0.74 | 82.85 | <1.0 ×10^-6^ | *51:02:01~16:04:01* | 0.0164 | 0.48 | 63.78 | <1.0 ×10^-6^ |
| *57:01:01~12:01:01* | 0.0033 | 0.49 | 20.36 | 6.0 × 10^-6^ | *52:01:01~12:01:01* | 0.0395 | 0.77 | 54.73 | <1.0 ×10^-6^ |
| *57:01:01~12:01:01* | 0.0033 | 0.25 | 8.53 | 3.0 × 10^-3^ | *52:01:01~12:02:01* | 0.0066 | 0.21 | 7.05 | 8.0 × 10^-3^ |
| *58:01:01~13:01:01* | 0.0066 | 0.27 | 5.11 | 0.02 | *53:01:01~04:01:01* | 0.0132 | 0.73 | 8.03 | 4.0 × 10^-3^ |
| *73:01~04:01:01* | 0.0099 | 0.71 | 13.57 | 2.0 × 10^-4^ | *55:01:01~01:02:01* | 0.0033 | 0.49 | 36.75 | <1.0 ×10^-6^ |
| ***B~C*** |  |  |  |  | *55:01:01~03:02:01* | 0.0099 | 0.73 | 34.72 | <1.0 ×10^-6^ |
| *07:02:01~15:02:01* | 0.0197 | 0.43 | 42.32 | <1.0 ×10^-6^ | *57:01:01~07:01:01* | 0.0132 | 0.46 | 5.78 | 0.02 |
| *08:01:01~07:01:01* | 0.0559 | 0.93 | 63.29 | <1.0 ×10^-6^ | *57:01:01~01:02:01* | 0.0033 | 0.49 | 20.36 | 6.0 × 10^-6^ |
| *13:01:01~06:02:01* | 0.0296 | 1.00 | 84.70 | <1.0 ×10^-6^ | *58:01:01~03:02:01* | 0.0099 | 0.47 | 21.35 | 4.0 × 10^-6^ |
| *15:10~07:01:01* | 0.0230 | 0.82 | 22.30 | 2.0 × 10^-6^ | *73:01~15:02:01* | 0.0099 | 0.73 | 36.99 | <1.0 ×10^-6^ |
| *18:01:01~12:01:01* | 0.0493 | 0.53 | 45.61 | <1.0 ×10^-6^ | ***DRB1~DQB1*** |  |  |  | <1.0 ×10^-6^ |
| *27:01~02:02:01* | 0.0099 | 0.59 | 88.47 | <1.0 ×10^-6^ | *01:01:01~05:01:01* | 0.0428 | 0.84 | 59.61 | <1.0 ×10^-6^ |
| *35:01:02~04:01:01* | 0.2072 | 0.75 | 170.51 | <1.0 ×10^-6^ | *03:01:01~02:01:01* | 0.0789 | 0.95 | 138.72 | <1.0 ×10^-6^ |
| *37:01~06:02:01* | 0.0033 | 1.00 | 9.16 | 2.5 × 10^-4^ | *03:02:01~04:01:01* | 0.0066 | 0.66 | 65.54 | <1.0 ×10^-6^ |
| **Haplotype** | **Frequency** | **D’** | **χ^2^** | ***P*** | **Haplotype** | **Frequency** | **D’** | **χ^2^** | ***P*** |
| ***DRB1~DQB1*** |  |  |  |  | ***DRB1~DQB1*** |  |  |  |  |
| *03:02:01~03:03:02* | 0.0033 | 0.32 | 15.40 | 8.7 × 10^-6^ | *11:01:01~03:01:01* | 0.3092 | 0.83 | 139.70 | <1.0 ×10^-6^ |
| *04:01:01~03:02:01* | 0.0461 | 0.92 | 81.44 | <1.0 ×10^-6^ | *13:01:01~06:01:01* | 0.0493 | 0.52 | 37.60 | <1.0 ×10^-6^ |
| *07:01:01~02:01:01* | 0.0461 | 0.57 | 43.45 | <1.0 ×10^-6^ | *14:01:01~05:01:01* | 0.0461 | 0.92 | 71.35 | <1.0 ×10^-6^ |
| *07:01:01~03:03:02* | 0.0066 | 0.28 | 6.21 | 0.01 | *15:01:01~06:01:01* | 0.1053 | 0.90 | 161.82 | <1.0 ×10^-6^ |
| *10:01:01~05:01:01* | 0.0230 | 1.00 | 38.21 | <1.0 ×10^-6^ | *16:01:01~05:01:01* | 0.0263 | 1.00 | 43.82 | <1.0 ×10^-6^ |
