# Supplementary material for: HLA-A, -B, -C, -DRB1 and -DQB1 allele and haplotype frequencies in Lebanese and their relatedness to neighboring and distant populations
Source: BMC Genomics. 2022 Jun 20;23:456. doi: 10.1186/s12864-022-08682-7 (PMC9208108; doi:10.1186/s12864-022-08682-7)
Supplement: Supplementary file 4 — Additional file 4: Supplementary Table 4 Ewens-Watterson homozygosity test of neutrality. [file 12864_2022_8682_MOESM4_ESM.docx]

**Supplementary Table 4**

Ewens-Watterson homozygosity test of neutrality

| **HLA Locus** | **Observed F** | **Expected F** | **Normalized deviate of F (Fnd)** | ***P*** |
| --- | --- | --- | --- | --- |
| *A* | 0.113 | 0.206 | -1.2146 | 0.025 |
| *B* | 0.097 | 0.130 | -0.7452 | 0.203 |
| *C* | 0.149 | 0.249 | -1.0622 | 0.073 |
| *DRB1* | 0.172 | 0.249 | -0.8230 | 0.180 |
| *DQB1* | 0.270 | 0.447 | -1.1056 | 0.102 |
