# Supplementary material for: HLA-A, -B, -C, -DRB1 and -DQB1 allele and haplotype frequencies in Lebanese and their relatedness to neighboring and distant populations
Source: BMC Genomics. 2022 Jun 20;23:456. doi: 10.1186/s12864-022-08682-7 (PMC9208108; doi:10.1186/s12864-022-08682-7)
Supplement: Supplementary file 5 — Additional file 5: Supplementary Table 5 Populations used in the present work. [file 12864_2022_8682_MOESM5_ESM.docx]

**Supplementary Table 5**

**Populations used in the present work**

| **No** | **Populations** | **Symbols** | **Size** | **No** | **Populations** | **Symbols** | **Size** |
| --- | --- | --- | --- | --- | --- | --- | --- |
| **1** | **Lebanese-H (*current*)** | **Leb-H** | **152** | 35 | Lebanese-B | Leb-B | 191 |
| 2 | Albanians | Alb | 160 | 36 | Lebanese-KZ | Leb-Kz | 93 |
| 3 | Algerians | Alge | 97 | 37 | Lebanese-NS | Leb-Ns | 59 |
| 4 | Algiers | Alg | 102 | 38 | Lebanese-Y | Leb-Y | 75 |
| 5 | Ashkenazi-Jews | Ash-J | 132 | 39 | Libyans | Lib | 118 |
| 6 | Basques-A | Bas-A | 82 | 40 | Libyans-Jews | Lib-J | 119 |
| 7 | Basques-Arratia | Bas-Ar | 83 | 41 | Macedonians | Mac | 172 |
| 8 | Basques-B | Bas-B | 99 | 42 | Mandenka | Man | 200 |
| 9 | Berbers-Metalsa | Ber-Me | 99 | 43 | Moroccans | Mor | 96 |
| 10 | Berbers-Zrawa | Ber-Z | 70 | 44 | Moroccans-A | Mor-A | 110 |
| 11 | Bubi | Bub | 101 | 45 | Moroccans-Agadir | Mor-Ag | 98 |
| 12 | Cretans | Cre | 135 | 46 | Moroccans-Chaouya | Mor-C | 98 |
| 13 | Egyptians | Egy | 101 | 47 | Moroccans-Jews | Mor-J | 94 |
| 14 | Egyptians-A | Egy-A | 121 | 48 | Mossi | Mos | 42 |
| 15 | Emiratis | Emi | 373 | 49 | Murcians | Mur | 173 |
| 16 | French | Fre | 179 | 50 | Omanis | Oma | 118 |
| 17 | French-Rennes | Fre-R | 200 | 51 | Palestinians | Pal | 165 |
| 18 | Fulani | Ful | 38 | 52 | Rimaibe | Rim | 39 |
| 19 | Gabesians | Gab | 77 | 53 | Saudis | Sau | 105 |
| 20 | Ghannouchians | Gha | 82 | 54 | Saudis-A | Sau-A | 213 |
| 21 | Greeks-A | Gre-A | 101 | 55 | Saudis-B | Sau-B | 158 |
| 22 | Greeks-B | Gre-B | 98 | 56 | Saudis-C | Sau-C | 499 |
| 23 | Greeks-C | Gre-C | 242 | 57 | Saudis-D | Sau-D | 383 |
| 24 | Greeks-D | Gre-D | 242 | 58 | Southern Tunisians | Tun-S | 250 |
| 25 | Iranian-Jews | Ira-J | 91 | 59 | Spaniards | Spa | 176 |
| 26 | Iranians | Ira | 120 | 60 | Syrians-A | Syr-A | 225 |
| 27 | Iranians-Azeri | Ira-Az | 100 | 61 | Tunisians | Tun | 376 |
| 28 | Iranians-Kurd | Ira-k | 100 | 62 | Tunisians-A | Tun-A | 80 |
| 29 | Iraq kurds | Iraq-K | 209 | 63 | Tunisians-C | Tun-C | 100 |
| 30 | Italians | Ita | 284 | 64 | Tunisians-M | Tun-M | 123 |
| 31 | Jordanians-A | Jor-A | 1254 | 65 | Turks | Tur | 250 |
| 32 | Lebanese | Leb | 95 | 66 | Turks-A | Tur-A | 228 |
| 33 | Lebanese-A | Leb-A | 1123 | 67 | Yemenite-Jews | Yem-J | 76 |
| 34 | Lebanese-Armen | Leb-Ar | 368 |  |  |  |  |
